# Targeted deletion of *SAP1* abolishes the expression of infectivity factors necessary for successful malaria parasite liver infection

**DOI:** 10.1111/j.1365-2958.2008.06271.x

**Published:** 2008-05-13

**Authors:** Ahmed S I Aly, Sebastian A Mikolajczak, Hilda Silva Rivera, Nelly Camargo, Vanessa Jacobs-Lorena, Mehdi Labaied, Isabelle Coppens, Stefan H I Kappe

**Affiliations:** 1 Seattle Biomedical Research InstituteSeattle, WA 98109, USA; 2 Department of Global Health, University of WashingtonSeattle, WA 98195, USA; 3 Department of Molecular Microbiology and Immunology, Malaria Research Institute, Johns Hopkins University Bloomberg School of Public HealthBaltimore, MD 21205, USA

## Abstract

Malaria parasite sporozoites prepare for transmission to a mammalian host by upregulation of *UIS* (Upregulated in Infectious Sporozoites) genes. A number of *UIS* gene products are essential for the establishment of the intrahepatocytic niche. However, the factors that regulate the expression of genes involved in gain of infectivity for the liver are unknown. Herein, we show that a conserved *Plasmodium* sporozoite low-complexity asparagine-rich protein, SAP1 (Sporozoite Asparagine-rich Protein 1), has an essential role in malaria parasite liver infection*.* Targeted deletion of *SAP1* in the rodent malaria parasite *Plasmodium yoelii* generated mutant parasites that traverse and invade hepatocytes normally but cannot initiate liver-stage development *in vitro* and *in vivo.* Moreover, immunizations with *Pysap1(−)* sporozoites confer long-lasting sterile protection against wild-type sporozoite infection. Strikingly, lack of SAP1 abolished expression of essential *UIS* genes including *UIS3*, *UIS4* and *P52* but not the constitutively expressed genes encoding, among others, sporozoite proteins CSP and TRAP. SAP1 localization to the cell interior but not the nucleus of sporozoites suggests its involvement in a post-transcriptional mechanism of gene expression control. These findings demonstrate that SAP1 is essential for liver infection possibly by functioning as a selective regulator controlling the expression of infectivity-associated parasite effector genes.

## Introduction

The first step of malaria transmission is the injection of sporozoites into a mammalian host by an anopheline mosquito bite (Vanderberg and Frevert, 2004; Amino *et al*., 2006). Initially, sporozoites form in mosquito midgut oocysts and subsequently invade and reside inside the salivary glands (reviewed in Matuschewski, 2006). In the mosquito salivary glands, sporozoites gain infectivity that is critical to support their transmission and life cycle progression in the mammalian liver (Vanderberg, 1974; 1975). Previous work has demonstrated that gain of infectivity is accompanied by extensive differential upregulation of unique gene products called UIS (Upregulated in Infectious Sporozoites) (Matuschewski *et al*., 2002). Indeed, *UIS* genes were shown to be essential for malaria parasite liver infection. *UIS3* and *UIS4* (Mueller *et al*., 2005a,b; Tarun *et al*., 2007) are proteins of the parasitophorous vacuole membrane (PVM), the principal host–parasite interface during cell infection (Mueller *et al*., 2005b; Mikolajczak *et al*., 2007a). Deletion of *UIS3* and *UIS4* leads to complete early arrest of liver-stage development inside the PVM (Mueller *et al*., 2005a,b; Tarun *et al*., 2007). Recently, it was shown that UIS3 interacts with liver fatty acid-binding protein (L-FABP) indicating a potential role of this protein in fatty acid uptake from the host hepatocyte (Mikolajczak *et al*., 2007a). Simultaneous deletion of the *UIS* gene *P52* (also called *P36p*), a putative GPI-anchored protein, and a non-*UIS* gene, *P36*, a putative secreted protein, renders sporozoites unable to form a PVM during infection and leads to complete developmental arrest at the early stage of hepatocyte infection (van Dijk *et al*., 2005; Ishino *et al*., 2005a; Labaied *et al*., 2007). Therefore, a number of UIS proteins critically contribute to establishing the intracellular parasitic niche either by the formation or modification of the host– parasite interface (reviewed in Mikolajczak and Kappe, 2006). However, it remains unknown what factors regulate the expression of *UIS* genes and consequently liver infectivity of sporozoites. Herein, we have identified a cytoplasmic low-complexity asparagine-rich protein, SAP1 (Sporozoite Asparagine-rich Protein 1) that is essential for liver infection possibly by means of regulating the expression of effector proteins such as P52, UIS3 and UIS4. Targeted deletion of *PySAP1* generated mutant parasites that traverse host cells, invade hepatocytes and form a PVM but cannot initiate liver-stage development and consequently completely lose mammalian infectivity *in vivo*. Drastically reduced transcript levels of liver infection-associated *UIS* genes in *SAP1*-deficient sporozoites in combination with SAP1's putative cytoplasmic localization suggest a post-transcriptional regulation of gene expression function of SAP1 in malaria parasite liver infection.

## Results

### SAP1 is a conserved *Plasmodium* sporozoite protein with an asparagine-rich low-complexity domain

**Fig. 1:**
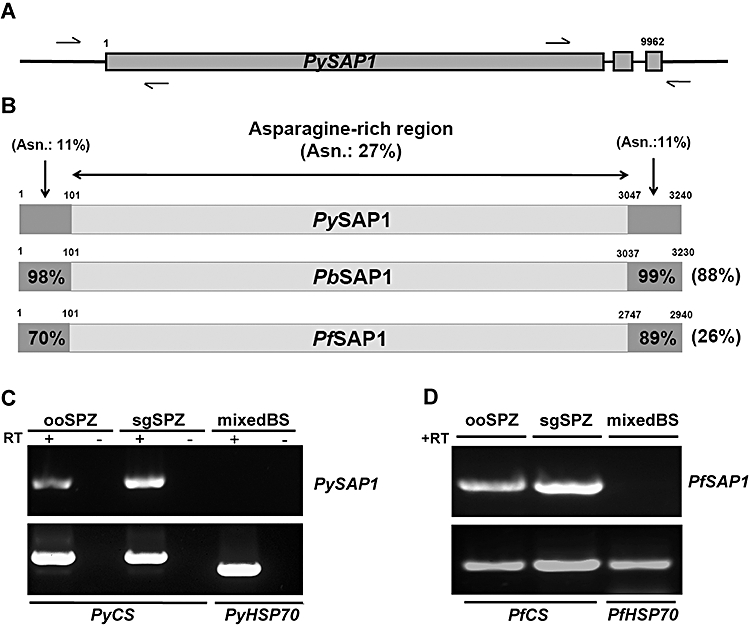
*SAP1* gene structure, protein structure, conservation among *Plasmodium* species and transcriptional profiling. A. A schematic representation of the *SAP1* gene organization: arrows show the locations of primers used for RT-PCR to identify the start and stop codons as well as exon 2 and exon 3. B. Alignment of the putative *Py*SAP1 with *Pb*SAP1 and *Pf*SAP1. The asparagine-rich regions are shown as light grey boxes bordered by non-asparagine-rich N- and C- termini shown as dark grey boxes. Total amino acid sequence identities to *Py*SAP1 are shown to the right and amino acid sequence identities between the N-termini and the C-termini are shown inside their respective boxes. Per cent (%) asparagine content is shown only for *Py*SAP1. C. RT-PCR analysis of RNA isolated from *P. yoelii* sporozoites shows the expression of *PySAP1* in ooSPZ (oocyst sporozoites) and sgSPZ (salivary gland sporozoites) but not in mixed blood stages (mixedBS). *PyCS* (circumsporozoite protein) is a positive RT-PCR control for sporozoite expression and *PyHSP70* is a positive RT-PCR control for mixed blood stages. D. RT-PCR analysis of different *P. falciparum* life cycle stages shows expression of *PfSAP1* in ooSPZ and sgSPZ but a lack of expression in blood stages (mixedBS).

We searched for putative cytoplasmic proteins that are highly expressed in sporozoites but not in blood stages because they might uniquely contribute to regulation of sporozoite infectivity. *SAP1* was first identified as a sporozoite-expressed gene in a suppression subtractive hybridization (SSH) screen of *Plasmodium yoelii* salivary gland sporozoites versus blood-stage merozoites (designated S22, sporozoite-specific gene 22) (Kaiser *et al*., 2004). *PySAP1* (gene identifier PY03269) has orthologues in other *Plasmodium* species including the human malaria parasite *P. falciparum* (gene identifier PF11_0480) (Carlton *et al*., 2002; Gardner *et al*., 2002). Alignment of *PfSAP1* and *PySAP1* revealed that the *PySAP1* open reading frame (ORF) was incomplete. Bioinformatics analysis and direct sequencing revealed that the *Py*SAP1 coding sequence was dispersed over two unassembled sequence contigs (described in Text S1 in *Supplementary material*). We confirmed the overlap between the two contigs encoding *Py*SAP1 by genomic PCR and reverse transcription polymerase chain reaction (RT-PCR) analysis. The correct start and stop codons as well as the correct exon–intron organization were also confirmed by RT-PCR analysis of salivary gland sporozoite RNA (Fig. 1A). The corrected *PySAP1* ORF nucleotide sequence (9723 nucleotides) and the predicted protein sequence (3240 amino acids) were deposited in NCBI GenBank (Accession No.: EU652769, Text S1). *PySAP1* encodes a large putative protein with a predicted 370 kDa molecular mass. *PySAP1* has one large exon followed by two small exons (Fig. 1A). Signal sequences, transmembrane domain(s), enzymatic or structural motifs were not identifiable in any of the predicted *Plasmodium*SAP1 protein sequences examined. SAP1 proteins are characterized by the presence of an extended internal asparagine-rich low-complexity domain, with an asparagine content of 27% in *P. yoelii* flanked by predicted globular domains with low asparagine content (Fig. 1B). Interestingly, these (N)- and (C)-terminal regions are highly conserved among *Plasmodium* species. The *Py*SAP1 N-terminus shares 70% amino acid sequence identity with the N-terminus of t*he P. falciparum* orthologue, and the *Py*SAP1 C-terminus shares 89% amino acid sequence identity with C-terminus of *PfSAP1* (Fig. 1B)*.* However, the overall amino acid sequence identity of SAP1 between *P. yoelii* and *P. falciparum* is only 26% due to the sequence divergence in the asparagine-rich domain (Fig. 1B).

### Sporozoite-specific expression profile of *PySAP1* and *PfSAP1*

RT-PCR analysis revealed that *PySAP1* is transcribed in oocyst and salivary gland sporozoites (Fig. 1C). As expected from the results of the previous SSH screen (Kaiser *et al*., 2004), no transcripts were detected in unsynchronized mixed blood stages (Fig. 1C). A similar expression pattern of *SAP1* was observed in *P. falciparum* oocyst and salivary gland sporozoites. No transcripts were detected in mixed blood stages. (Fig. 1D). Therefore, the sporozoite-specific expression profile of *PfSAP1* is similar to the expression profile of *PySAP1*.

### Localization of *Py*SAP1

**Fig. 2:**
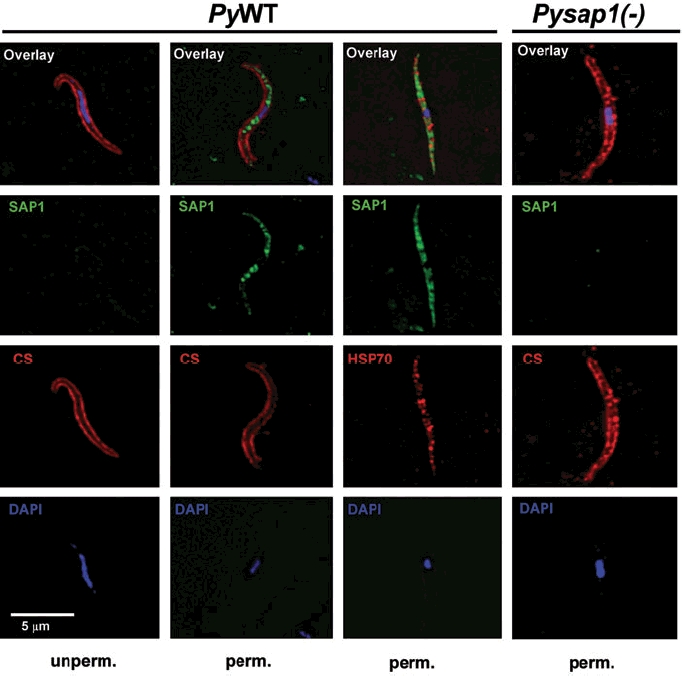
SAP1 is localized to the cell interior of *Plasmodium* sporozoites. Immunofluorescence assays on air-dried *Py*WT or *Pysap1*(*−)* salivary gland sporozoites show the predicted internal localization of SAP1 in *Py*WT, which is detected only after sporozoite permeabilization with saponin (perm.). SAP1 staining was not detected in *Pysap1*(*−)* sporozoites after sporozoite permeabilization. Note the absence of SAP1 staining in the nucleus of *Py*WT sporozoites. Scale bar is 5 μm.

To determine the cellular localization of SAP1, we generated rabbit polyclonal antisera against a peptide in the C-terminus of *PySAP1* and tested the antisera in immunoflourescence assays (IFAs) using *P. yoelii* sporozoites. A specific sporozoite-internal staining that excluded the nucleus and was distinct from circumsporozoite (CS) protein staining was observed (Fig. 2). SAP1 localization appeared uneven and clustered within the sporozoite cytoplasm. Its localization appeared also distinct when compared with cytoplasmic heat shock protein (HSP70) staining, which was induced by incubation of the sporozoites at 37°C for 1 h (Fig. 2). SAP1 staining was only observed in sporozoites after membrane permeabilization, indicating that SAP1 localizes exclusively to the interior of the sporozoite. Together, the data suggest that SAP1 localizes to the cytoplasm, intracellular organelles or other structures within the cytoplasm as predicted by the lack of a secretory signals in the SAP1 sequence. Pre-immune sera did not show reactivity with sporozoites (data not shown).

### Targeted deletion of *PySAP1* and knockout phenotype in blood and mosquito stages

**Fig. 3:**
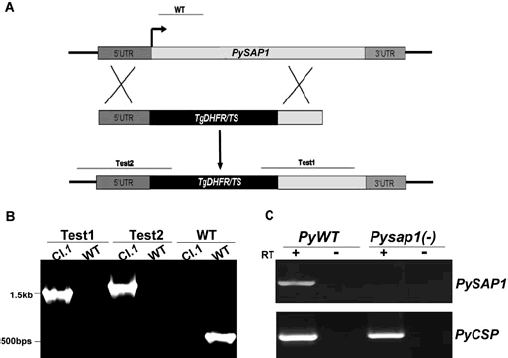
Targeted deletion of *PySAP1*. A. Schematic representation of the replacement strategy to generate *Pysap1*(*−)* parasites. The endogenous *PySAP1* genomic locus is targeted with a replacement fragment containing the 5′ and 3′ sequence within exon 1 of *PySAP1* flanking the *Toxoplasma gondii DHFR*/*TS*-positive selection marker. Diagnostic wild type-specific or integration-specific test amplicons are indicated by lines. B. PCR genotyping shows the gene replacement using oligonucleotide primer combinations that can only amplify from the recombinant locus (Test 1 and Test 2). The wild type-specific PCR reaction (WT) confirms the absence of wild-type parasites in the clonal *Pysap1*(*−)* parasites. C. RT-PCR analysis (35 cycles) shows the loss of *PySAP1* transcripts in RNA isolated from *Pysap1*(*−)* sgSPZs. The *PySAP1*-specific amplicon used for the analysis is shown above in (A) as the wild type (WT) test. *PyCSP* was used as a positive control.

Targeted gene deletion of *PySAP1* was conducted by double-cross-over homologous recombination to replace the majority of the coding sequence with the *TgDHFR/TS* selection marker cassette (Fig. 3A) (Menard and Janse, 1997). Deletion-specific genomic PCR analysis confirmed the successful double-cross-over recombination event and the successful isolation of a *Pysap1(−)* parasite clone with pure gene deletion background (Fig. 3B). Therefore, *PySAP1* was successfully deleted in the erythrocytic stages with no observed deficiency of blood-stage development (data not shown). In addition, the morphology of male and female gametocytes in thin infected-blood smears and male gamete exflagellation in wet mounts of infected blood were indistinguishable from *P. yoelii* wild-type (WT) parasites (data not shown). Transmission of *Pysap1(−)* parasites to mosquitoes resulted in normal midgut infection and oocyst development. *Pysap1(−)* oocyst sporozoites developed in a similar manner as *Py*WT oocyst sporozoites (Table S1 in *Supplementary material*). Importantly, *Pysap1(−)* sporozoites accumulated in the salivary glands in numbers comparable to WT, indicating normal salivary gland infection (Table S1). RT-PCR analysis confirmed the absence of *PySAP1* transcripts in *Pysap1(−)* sporozoites (Fig. 3C). IFAs with the anti-SAP1 antisera were negative, confirming that *Pysap1(−)* sporozoites do not express SAP1 (Fig. 2). The data give further support to the specificity of the SAP1 antisera. We conducted all initial experiments with two independent clones of *Pysap1(−)* that were identical in their phenotypes (data not shown). Thereafter, experiments were conducted with a single *Pysap1(−)* clone.

### *Pysap1(−)* sporozoites fail to induce blood-stage infection and elicit sterile protection against *Py*WT sporozoite challenge

We tested the infectivity of *PySAP1*-deficient salivary gland sporozoites in susceptible BALB/c mice. Mosquito bite experiments with more than 50 *Pysap1(−)*-infected mosquitoes/mouse did not result in blood-stage infection (data not shown). Strikingly, intravenous (iv) injection of escalating doses of *Pysap1(−)* salivary gland sporozoites did not lead to blood-stage parasitaemia, tested daily by blood smears until day 14 post infection (Table 1). Even with extremely high doses of more than 2 million sporozoites no subsequent blood-stage parasitaemia was observed. This highest dose corresponded to a ∼200 000-fold increase over the minimal infectious dose of *Py*WT sporozoites administered to BALB/c mice by iv injection (Belmonte *et al*., 2003). Hence, we conclude that *Py*SAP1 is essential for parasite pre-erythrocytic stage functions after transmission from the mosquito to the mammalian host.

**Table 1:** *PySAP1*-deficient sporozoites are completely attenuated and do not cause blood-stage infection in BALB/c mice.

	*Pysap1(−)*		*Py*WT	
		
No. of injected sporozoites	Infected	Pre-patent period^a^	Infected	Pre-patent period^a^
20	ND	ND	2/2	4 days
100	ND	ND	6/6	4 days
10 000	0/30	–	8/8	3 days
100 000	0/15	–	3/3	2.5 days
500 000	0/8	–	ND	ND
1 000 000	0/4	–	ND	ND
> 2 000 000	0/3	–	ND	ND

a The period (in days) between sporozoite infection and the detection of erythrocytic stages in blood smears.

ND, not done.

We next tested whether *Pysap1(−)* salivary gland sporozoite immunization of mice can induce sterile protection against *Py*WT sporozoite challenge. Four groups of BALB/c mice were immunized iv with three doses of 10 000 *Pysap1(−)* salivary gland sporozoites, in 2-week intervals (Table 2). The first immunization group (group I) was challenged by iv injection of 10 000 *Py*WT sporozoites at day 7 after the last immunization dose. Two of the immunization groups (groups II and III) were challenged by iv injection of 10 000 *Py*WT sporozoites 30 and 210 days after the last immunization dose. The mice of group III were then challenged by *Py*WT erythrocytic stages 2 weeks after the last challenge with either 10^3^ or 10^6^ asexual blood stages injected iv or intraperitoneally (ip) into five mice each respectively (data not shown). The fourth group (group IV) was challenged by infectious mosquito bite 45 and 210 days after the last immunization dose. All mice were protected when challenged with *Py*WT sporozoites and did not develop any blood-stage infection (Table 2). However, mice challenged with blood-stage parasites developed blood-stage parasitaemia after 2 days (data not shown). The data demonstrate that *Pysap1(−)* salivary gland sporozoite immunizations induce stage-specific sterile immunity against subsequent *Py*WT sporozoite infection but not against asexual blood-stage infection.

**Table 2:** Immunization with *Pysap1(−)* sporozoites confers sterile protection against wild-type sporozoite challenge.

Group	Primary dose (days of booster dose)	Challenge dose/days after last boost	No. protected/No. challenged^a^	Mean pre-patent period (days)
I	10 000 (14, 28)	10 000/7	9/9	−
II	10 000 (14, 28)	10 000/(30)/(210)	15/15/15	−/−
III	10 000 (14, 28)	10 000/(30)/(210)	10/10/10	−/−
IV	10 000 (14, 28)	MB^b^/(45)/(210)	5/5/5	−/−

a Each immunization group had an age-matched naïve control group (minimum three mice) that all became patent at day 3 after each *Py*WT sporozoite challenge.

b Infection through mosquito bite (MB) by allowing a minimum of 10 *Py*WT female infected mosquitoes, with midgut oocyst infectivity higher than 90%, to bite one mouse for at least 10 min.

### *Pysap1(−)* sporozoites traverse and invade hepatocytes normally but suffer an early liver-stage developmental arrest *in vitro*

**Fig. 4:**
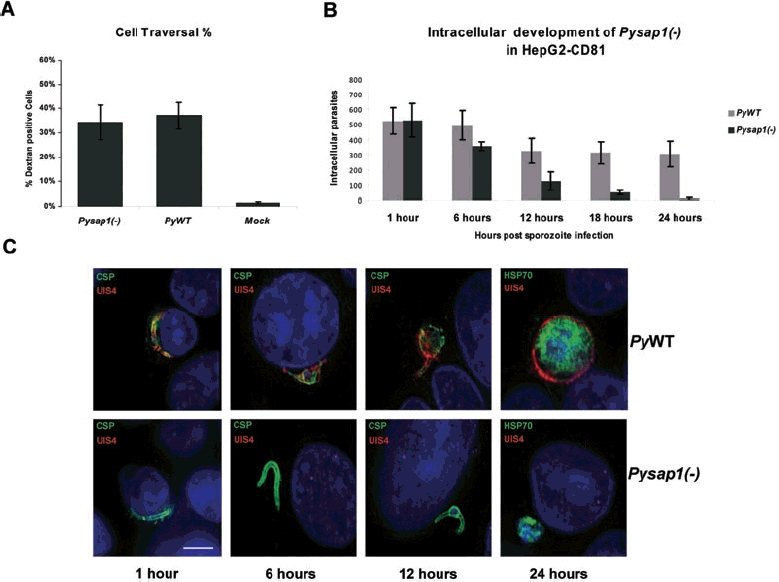
*Pysap1*(*−)* parasites traverse and invade hepatocytes but fail to complete liver-stage development *in vitro*. A. Graph shows the per cent of dextran-positive hepatoma cells as an indication of sporozoites cell-traversal activity. No significant difference can be detected between *Pysap1*(*−)* and wild type (*Py*WT). B. Graph shows the number of intracellular liver stages, determined by differential permeabilization, at different time points post sporozoite infection. The number of *Pysap1*(*−)* intracellular parasites is similar to *Py*WT at 1 and 6 h post infection but drastically decreases at 12 and 18 h. At 24 h virtually no *Pysap1*(*−)* liver stages survived. Note that the number of WT liver stages in this assay initially decreases but stabilizes after 12 h. C. Immunofluorescence assays show liver-stage development at different time points after sporozoite infection. *Pysap1*(*−)* intracellular parasites (CSP and HSP70 staining in green) do not express the PVM marker UIS4 (red) and arrest in development. Growth-arrested *Pysap1*(*−)* liver stages shown at 24 h post infection were detected rarely in this assay. Scale bar is 5 μm.

Failure of mutant salivary gland sporozoites to induce blood-stage infection in mice can be due to distinct knockout phenotypes (reviewed in Mikolajczak and Kappe, 2006). *Pysap1(−)* salivary gland sporozoites displayed continuous gliding motility that was undistinguishable from *Py*WT, tested on glass slides by direct microscopic examination (Vanderberg, 1974) (data not shown). Thereafter, we tested the cell-traversal capacity of *Pysap1(−)* salivary gland sporozoites using a cell-wounding assay (Vanderberg *et al*., 1990; Mota *et al*., 2001). *Pysap1(−)* sporozoites traversed hepatocytes and wounded cells at a rate comparable to *Py*WT sporozoites (Fig. 4A). In order to identify and characterize the deficiency of *Pysap1(−)* sporozoites in completing pre-erythrocytic infection, we conducted *in vitro* assays with the hepatoma cell line HepG2-CD81 which sustains productive *P. yoelii* sporozoite infection and liver-stage development (Silvie *et al*., 2006). Intrahepatocytic parasites were quantified by differential permeabilization at 1, 6, 12, 18 and 24 h post *in vitro* infection (pi) using fluorescence microscopy. Interestingly, at 1 h pi the number of *Pysap1(−)* intracellular parasites was similar to *Py*WT infections, but *Pysap1(−)* intracellular liver-stage numbers gradually decreased in comparison with *Py*WT infections at 6 h and 12 h pi (Fig. 4B). *Pysap1(−)* intracellular parasite numbers then sharply decreased at 18 h pi and at 24 h pi there were almost no intracellular parasites, when compared with *Py*WT (Fig. 4B). Most importantly, intrahepatocytic *Pysap1(−)* parasites failed to grow and develop. They appeared smaller and deficient in their transformation to liver-stage trophozoites when compared with *Py*WT liver stages (Fig. 4C). Surprisingly, immunostaining of the PVM-resident protein UIS4 showed that it was not detected in *Pysap1(−)* parasites at all time points tested, whereas in *Py*WT liver stages a UIS4 positive PVM staining pattern was observed at all time points later than 1 h pi (Fig. 4C). Evidently, *Pysap1(−)* sporozoites infect host hepatocytes but suffer growth arrest early during liver-stage development *in vitro*, suggesting that an early growth defect causes the lack of *Pysap1(−)* sporozoite infectivity to mice.

### Electron microscopic analysis of intrahepatocytic *Pysap1(−)* sporozoites

**Fig. 5:**
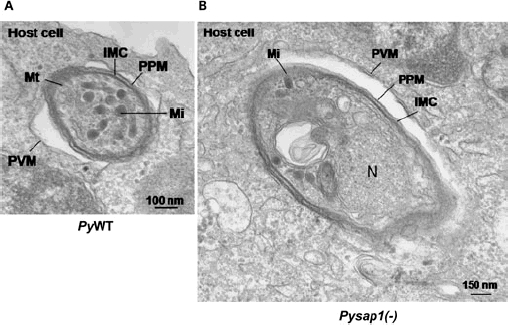
*Pysap1*(*−)* liver stages form a parasitophorous vacuole membrane (PVM). Electron microscopic analysis establishes that intrahepatocytic *Pysap1*(*−)* parasites form a PVM. A. Transversal section of a *Py*WT parasite within a HepG2-CD81 cell 1 h post infection. The PVM is indicated. B. Transversal section of *Pysap1*(*−)* sporozoite 1 h post infection in HepG2-CD81. The PVM is indicated. IMC, inner membrane complex; Mi, microneme; N, nucleus; PPM, parasite plasma membrane; PVM, parasitophorous vacuole membrane; Mt, mitochondrion.

Intracellular malaria parasites need a PVM for development (reviewed in Mikolajczak and Kappe, 2006). Therefore, we examined whether the observed lack of UIS4 in intracellular *Pysap1(−)* parasites indicated a possible deficiency in PVM formation. We performed an electron microscopic analysis of intracellular WT and *Pysap1(−)* parasites 1 h after infection of HepG2-CD81 cells. Intracellular *Pysap1(−)* parasites were able to form a PVM (Fig. 5). Out of 15 intrahepatocytic *Pysap1(−)* parasites evaluated by EM, 4 exhibited a PVM and 11 appeared free in the cytoplasm. The latter may represent sporozoites in the process of cell traversal. However, it is also possible that *Pysap1(−)* sporozoites form a PVM but less efficiently than *Py*WT.

### *UIS* gene products are depleted in *Pysap1(−)* sporozoites

**Fig. 6:**
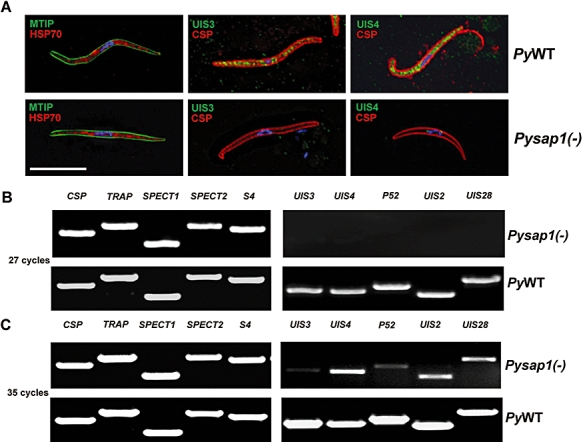
Essential UIS proteins are depleted in *Pysap1*(*−)* sporozoites. A. Immunofluorescence assays on air-dried *Py*WT salivary gland sporozoites show expression of CSP and HSP70 (red), MTIP, UIS3 and UIS4 (green). *Pysap1*(*−)* sporozoites exhibit MTIP, HSP70 and CSP staining but lack UIS3 and UIS4. Scale bar is 5 μm. B. Twenty-seven-cycle non-quantitative RT-PCR analysis with RNA isolated from *Py*WT and *Pysap1*(*−)* salivary gland sporozoites showing expression of *UIS* transcripts in WT sporozoites and the depletion of *UIS* transcripts in *Pysap1*(*−)* sporozoites (right). Transcripts of non-UIS genes that are essential for sporozoites prior to or in hepatocyte infection appear unaffected (left). C. Thirty-five-cycle non-quantitative RT-PCR analysis, with the same RNA used in (B). At higher PCR cycle number UIS transcripts are detected.

Despite of the presence of a PVM we noted the lack of *Py*UIS4 in *Pysap1(−)* liver stages (Fig. 4C). *Py*UIS4 is normally expressed in sporozoite secretory organelles as well as the liver-stage PVM and is essential for malaria parasite liver-stage development (Mueller *et al*., 2005b). To test whether *Py*UIS4, as well as other proteins, is expressed in *Pysap1(−)* sporozoites prior to hepatocyte invasion, we performed IFAs to test UIS4, UIS3 and MTIP (Bergman *et al*., 2003) expression in *Pysap1(−)* and *Py*WT salivary gland sporozoites. In *Py*WT sporozoites we detected expression of each protein (Fig. 6A). In contrast, we did not detect expression of UIS4 or UIS3 in *Pysap1(−)* sporozoites but did detect MTIP staining (Fig. 6A). To test whether this expression pattern is due to a reduction in *UIS4* and *UIS3* transcript abundance, which would potentially indicate transcript degradation (Parker and Sheth, 2007), we performed RT-PCR analysis on *Pysap1(−)* salivary gland sporozoite cDNA. Strikingly, we observed a severe reduction of *PyUIS4* and *PyUIS3* (Mueller *et al*., 2005a,b) transcript abundance (Fig. 6B and C). Furthermore, we saw a decrease in *P52* (van Dijk *et al*., 2005; Ishino *et al*., 2005a,b; Labaied *et al*., 2007) transcript abundance as well as the transcripts of two uncharacterized UIS genes, *UIS2* (putative secreted phosphatase) and *UIS28* (putative secreted lipase) (Matuschewski *et al*., 2002*)* (Fig. 6B and C). Conversely, transcript abundance for genes that are involved in sporozoite functions prior to PVM formation and liver-stage development appeared not significantly reduced in *Pysap1(−)* sporozoites (Fig. 6B and C). These genes included *CSP*, *TRAP* (reviewed in Kappe *et al*., 2004; Baldacci and Menard, 2004), *SPECT1* (Ishino *et al*., 2004), *SPECT2* (Ishino *et al*., 2005b) and S4/*CELTOS* (Kaiser *et al*., 2004, Kariu *et al*., 2006). Therefore, lack of *Py*SAP1 in sporozoites has a selective negative impact on *UIS* gene expression in *P. yoelii*.

## Discussion

Successful hepatocyte infection and liver-stage development by the malaria parasite is dependent on establishment of a PVM as the functional host–parasite interface. Some *UIS* gene products uniquely expressed in salivary gland sporozoites and localized to the PVM in liver stages play essential roles in intrahepatocytic parasite survival (Mueller *et al*., 2005a,b; Mikolajczak *et al.*, 2007a; Tarun *et al*., 2007). However, factors that allow salivary gland sporozoites lying-in-wait in the mosquito salivary glands to initiate a co-ordinated switch to mammalian host infection by differential expression of *UIS* have not been identified. Our work identifies SAP1 as such a potential factor. *Py*SAP1 is the first identified cytoplasmic *Plasmodium* protein with an essential function for pre-erythrocytic stages. It has a large internal asparagine-rich low-complexity domain. Low-complexity domains are frequently found in *Plasmodium* proteins (Aravind *et al*., 2003). This is partially the consequence of the high A/T nucleotide content in the *Plasmodium* genome (Pizzi and Frontali, 2001; Singh *et al*., 2004). Low-complexity domains in *Plasmodium* proteins were hypothesized to be an evolutionary by-product with no significant function in the biology of the malaria parasite (Xue and Forsdyke, 2003). In contrast, low-complexity proteins have been proposed as virulence inducing factors in some pathogenic bacterial strains (Nandi *et al*., 2003). The low-complexity domain of SAP1 is flanked by two highly conserved non-asparagine-rich N- and C-terminal domains. The level of conservation in these domains is high among SAP1 proteins from distinct *Plasmodium* species, indicating that they represent functionally important regions. *Pysap1(−)* salivary gland sporozoites show extremely reduced transcript abundance for *UIS3*, *UIS4* and *P52* but not *SPECT*s, *TRAP* and *CSP*, indicating a selective mechanism of *UIS* transcript depletion. It will be interesting to determine whether transcript abundance for additional genes is affected in the *Pysap1(−)* sporozoites as we have shown here for the uncharacterized *UIS2* and *UIS28*. Clearly, lack of *UIS* genes expression cannot be attributed to a defect in salivary gland invasion and residence as *Pysap1(−)* sporozoites infected the salivary glands with efficiencies that are comparable to WT sporozoites. Therefore, it appears likely that the reduction of *UIS* transcript expression in *Pysap1(−)* sporozoites is a direct effect of the lack of SAP1 and not an indirect effect of an altered biological behaviour of the *Pysap1(−)* mutants. Transcript abundance in eukaryotes is mainly regulated by transcriptional and post-transcriptional mechanisms. *Py*SAP1 localization to the sporozoite cytoplasm and absence from the sporozoite nucleus suggests that *Py*SAP1 is involved in as-yet-to-be-defined post-transcriptional mechanisms of UIS transcript regulation, as post-transcriptional regulation is expected to be executed in the cytoplasm of the cell (Elemento *et al*., 2007; Parker and Sheth, 2007). However, the C-terminus of *Py*SAP1, which is recognized by the antisera, might be processed and left in the cytoplasm. In this scenario, the remaining part of the protein might translocate to the nucleus, where it might exert its effect by contributing to the regulation of transcription of *UIS* genes. Interestingly, post-transcriptional, but not transcriptional, regulation has been hypothesized to be the main pathway for controlling the expression levels of proteins in *Plasmodium* (Coulson *et al*., 2004; Hakimi and Deitsch, 2007). Indeed, it has been suggested that *Plasmodium* must rely on this mechanism for controlling the extensive differential gene expression required during the complex malaria parasite life cycle (Hall *et al*., 2005). Recently, it has been shown that a RNA helicase termed DOZI (Development of Zygote Inhibited) is expressed in the female gametocyte where it localizes to cytoplasmic protein complexes and is involved in translational repression of transcripts (Mair *et al*., 2006). *DOZI* knockouts showed a severe reduction in the levels of many sexual stage-specific transcripts, presumably because they were subject to rapid degradation when not protected in ribonucleoprotein (RNP) complexes. We speculate that SAP1 might be an essential component of such an RNP complex in sporozoites and protects UIS transcripts specifically from degradation. This scenario however requires further investigation. Interestingly, proteins with glutamine and asparagine-rich domains have recently been shown to be part of RNP complexes in yeast where they act as scaffolding proteins (Decker *et al*., 2007).

*Pysap1(−)* sporozoites showed complete attenuation of liver infection. This is clearly attributable to the lack of the essential proteins UIS3, UIS4 and P52 and possibly additional UIS in the knockout parasite and likely not to a lack of a direct effector function of *Py*SAP1. Single- and double-gene-deletion sporozoites are effective live attenuated vaccines in mouse models (reviewed in Mikolajczak *et al*., 2007b) and we have shown herein that *Pysap1(−)* sporozoites also confer sterile long-lasting protection against *Py*WT sporozoite challenge. *P. falciparum* gene deletion mutants might go forward for testing as human malaria vaccines (Renia *et al*., 2006). We suggest that a putative *P. falciparum sap1(−)* sporozoite may be an attractive live attenuated vaccine candidate due to its quasi-multilocus attenuation. Together, our data give initial insights into the regulation of malaria parasite infectivity after mosquito transmission and these findings might advance efforts to develop measures for prevention of malaria infection.

## Experimental procedures

### Experimental animals, parasites and cell lines

Six- to 8-week-old female BALB/cJ (for *in vivo* infection studies and immunizations) or Swiss Webster (SW) mice (for parasite cycle maintenance) were purchased from the Jackson Laboratory (Bar Harbor, ME) or Harlan (Indianpolis, IN). Animal handling was conducted according to Institutional Animal Care and Use Committee-approved protocols. Wild-type *P. yoelii* 17XNL (non-lethal strain) clone 1.1 (Weiss *et al*., 1989*)* and *Pysap1(−)* parasites were cycled between SW mice and *Anopheles stephensi* mosquitoes. Infected mosquitoes were maintained on sugar water at 24°C and 70% humidity. Salivary gland sporozoites were extracted from infected mosquitoes between days 13 and 15 post blood meal infection as described before (Labaied *et al*., 2007; Tarun *et al*., 2007). The human hepatoma cell line HepG2-CD81 (Silvie *et al*., 2006) was used for all *in vitro* assays and was maintained in DMEM-F12 medium supplemented with antibiotics and 10% fetal calf serum (FCS).

### Generation of *Pysap1(−)* parasites

Targeted deletion of *PySAP1* by double-cross-over homologous recombination was achieved constructing a replacement plasmid in the b3D.DT.H Db targeting vector (Janse *et al*., 2006; Jongco *et al*., 2006). *P. yoelii* 17XNL genomic DNA (gDNA) was used as a template to amplify a 1.5 kb fragment of the 5′UTR of *PySAP1* using oligonucleotide primers PySAP1rep1 forward (F) and PySAP1rep2 reverse (R) (primers sequences provided in Table S2). The amplified fragment was inserted into the transfection plasmid between KpnI and HindIII restriction enzymes sites. Similarly, a 1 kb DNA fragment from the 3′ORF sequence of *PySAP1* was amplified using PySAP1rep3F and PySAP1rep4R primers and inserted between the SpeI and SacII restriction sites in the transfection vector. The resulting plasmids were digested with KpnI and SacII to release the replacement fragment used for the transfection. Transfection of *P. yoelii* 17XNL parasites using the Amaxa nucleofector device (Amaxa GmbH, Germany), resistant parasites selection and recombinant parasite cloning by serial limiting dilutions were all conducted as described elsewhere (Janse *et al*., 2006; Jongco *et al*., 2006). We obtained two independent *Pysap1(−)* clonal parasite populations that were phenotypically identical. Detailed analysis was performed with one representative clone. To confirm the targeted deletion and the new genetic recombination, integration-specific gDNA PCR amplification of the *Pysap1(−)* locus was generated using the specific primers combinations Test1 (TgF and PySAP1TestR) and Test2 (PySAP1TestF and TgR) (primers sequences provided in Table S2). Whereas *PySAP1* locus-specific gDNA-PCR amplification was generated using the primers combination WT (PySAP1orfF and PySAP1orfR), the primers combination test for the WT *PySAP1* locus was also used in RT-PCR to confirm the absence of the *PySAP1* transcript from *Pysap1(−)* recombinant parasite clone.

### Reverse transcription polymerase chain reaction (RT-PCR)

Total RNA was extracted using TRIzol reagent (Invitrogen) and treated with TURBO DNase (Ambion) from *P. yoelii* salivary gland sporozoites (2 × 10^6^), oocyst sporozoites (2 × 10^6^) or mixed blood stages (1 × 10^7^ infected red blood cells were isolated from an infected mouse by heart puncture bleeding and serial limiting dilution of infected blood). *P. falciparum* sporozoites and mixed blood-stage RNA materials were kindly provided by Dr Urszula Krzych and Dr Jack Williams (WRAIR, Silver Spring, MD). cDNA synthesis was performed using the Super Script III Platinum two-step qRT-PCR kit (Invitrogen). The sequences of specific primers used for amplification from cDNA are listed in Table S2. All PCR amplification cycles were performed at 95°C for 30 s for DNA denaturation, 55°C for 30 s for primer annealing, 60°C for 4 min for DNA strands extension.

### Immunofluorescence assays (IFAs)

We generated rabbit polyclonal antiserum against a *Py*SAP1_3020−3034_ synthetic peptide (LRGRQVQQSFNHSAS). The antiserum was further affinity-purified against the synthetic peptide and the specific IgGs were concentrated. *Pysap1(−)* or *Py*WT sporozoites were air dried on poly-lysine-treated glass slides after incubation for 1 h at 37°C. Sporozoites were fixed with 4% paraformaldehyde (PFA) for 10 min at room temperature. This was followed by permeabilization with 0.2% saponin for 15 min at room temperature. After blocking in 10% FCS/PBS overnight (ON) at 4°C, primary antibodies were diluted 1:300 in 10%FCS/PBS and incubated with the sporozoites for 1 h at 37°C. Mouse monoclonal antibodies 2F6 (Potocnjak *et al*., 1980; Yoshida *et al*., 1980*)* and 2E6 (Tsuji *et al*., 1994*)* were used against *Py*CSP and *Py*HSP70, respectively, and rabbit polyclonal antisera against *Py*SAP1, *Py*UIS4, *Py*UIS3, *Py*MTIP were used in sporozoite IFAs. Alexa Fluor (Molecular Probes, Eugene, OR) conjugated secondary antibodies were also diluted in 10%FCS/PBS and incubated with the sporozoites for 1 h at 37°C. Conjugated anti-rabbit Alexa Fluor 488 (green) and anti-mouse Alexa Fluor 594 (red) were used to visualize the bound primary antibodies. Nuclear staining with 4′,6′-diamidino-2-phenylindole (DAPI) was conducted within the last washing step with PBS (1:2000) and before mounting the slides with an antifade reagent (Fluoroguard; Bio-Rad, Hercules, CA). Preparations were analysed using fluorescence confocal microscopy (Olympus 1 × 70 Delta Vision).

### Mouse infections and immunizations

For sporozoite immunizations and challenges, BALB/cJ mice were injected iv with sporozoites re-suspended in incomplete DMEM-F12 medium. Blood-stage patency was monitored daily by evaluation of Giemsa-stained blood smears from day 2 to day 14 post sporozoite infection. For blood-stage challenge experiments, immunized mice that had been challenged with sporozoites twice were injected either ip with 10^6^ or iv with 10^3^*Py*WT erythrocytic asexual stages in RPMI. *Py*WT blood stages were isolated from an infected mouse by heart puncture followed by serial limited dilution.

### Cell-traversal assay

Hepatoma HepG2-CD81 cells were inoculated in eight-well chamber slides at a density of 60 000 cells well^−1^ 2 days before the assay. A total of 20 000 sporozoites per well of *Py*WT or *Pysap1(−)*, in addition to uninfected mosquitoes salivary gland extracts (mock), were re-suspended in incomplete DMEM-F12 medium with 3% bovine albumin serum (BSA) and 2 mg ml^−1^ fluorescein isothiocyanate (FITC)-dextran (Invitrogen-Molecular Probes, Eugene, OR). Sporozoites and mock suspensions were added to the cells, centrifuged for 2 min at 1000 r.p.m. and incubated for 1 h at 37°C. Thereafter, the cells were washed thoroughly with PBS and complete DMEM medium twice to remove any extracellular dextran, and the cells were allowed to grow for a further 3 h in complete DMEM medium. Flow cytometric quantitative analysis of dextran-positive cells was conducted using a flow cytometer (Cytopia, Seattle, WA) and the flow cytometry analysis program FlowJo version 7.0.3 (TreeStar, Ashland, OR) (Labaied *et al*., 2007).

### *In vitro* infection assays

We standardized a differential permeabilization hepatoma infection assay to specifically quantify liver-stages parasites at different time points of infection by fluorescence microscopy. Hepatoma HepG2-CD81 cells were seeded in eight-well chamber slides at a density of 40 000–50 000 cells well^−1^ 2 days before the assay. A total of 50 000 sporozoites of *Pysap1(−)* or *Py*WT re-suspended in incomplete DMEM-F12 medium were added per well. The sporozoites were incubated for 1 h with the cells at 37°C. The cells were washed with PBS and with complete DMEM F12 media twice to remove all non-invading and unbound sporozoites and mosquito debris. One hour post infection assays were fixed with 4% PFA (which does not permeabilize hepatocytes) for 10 min at room temperature, followed by permeabilization (or not) with ice-cold methanol for 5 min at room temperature and then blocking in 10%FCS/PBS (ON) at 4°C. The cells for other post-infection time points assays were further grown in complete DMEM medium until fixed, permeabilized (or not) and blocked at 6 h, 12 h, 18 h and 24 h. Primary antibodies against *Py*CSP, *Py*HSP70 and *Py*UIS4 were diluted to 1:300 in 10%FCS/PBS and incubated with the cells for 1 h at 37°C. Conjugated secondary anti-mouse Alexa Fluor 488 (green) and anti-rabbit Alexa Fluor 594 (red) were used to visualize the bound primary antibodies. Nuclear staining with DAPI (1:3000 diluted) was performed with the last wash and before mounting the slides with an antifade reagent. Preparations were analysed using fluorescence confocal microscopy (Olympus 1 × 70 Delta Vision). Intracellular parasites were determined as the total number of parasites counted in each permeabilized sample well as a fraction of the total number of parasites counted in the control unpermeabilized sample well.

### Transmission electron microscopy

For thin-section transmission electron microscopy, 10^6^*Py*WT and *Pysap1(−)* sporozoites were used to infect 10^6^ subconfluent HepG2-CD81 cells. One hour post infection, cells were fixed with 2.5% glutaraldehyde (Electron Microscopy Sciences, Hatfield, PA) in 0.1 M sodium cacodylate buffer (pH 7.4) for 1 h at room temperature and processed as described previously (Quittnat *et al*., 2004), before examination with a Philips 410 electron microscope (Eindhoven, the Netherlands) under 80 kV.

## Supplementary material

This material is available as part of the online article from: http://www.blackwell-synergy.com/doi/abs/10.1111/

j.1365-2958.2008.06271.x

(This link will take you to the article abstract).
