# Supplementary material for: Targeted deletion of SAP1 abolishes the expression of infectivity factors necessary for successful malaria parasite liver infection
Source: Mol Microbiol. 2008 May 13;69(1):152–63. doi: 10.1111/j.1365-2958.2008.06271.x (PMC2615191; doi:10.1111/j.1365-2958.2008.06271.x)
Supplement: Supplementary file 1 [file mmi0069-0152-SD1.pdf]

**Supplemental Table 1. Depletion of *PySAP1* does not affect sporozoite development in *Anopheles* mosquitoes.**

| Experiment | Genotype | Mean ooSPZs/ mosquito[1] | Mean sgSPZs/ mosquito[2] |
|---|---|---|---|
| 1 | *Pysap1(-)* | 79,000 | 17,000 |
| | *Py*WT | 85,000 | 18,000 |
| 2 | *Pysap1(-)* | 96,000 | 34,000 |
| | *Py*WT | 102,000 | 27,000 |
| 3 | *Pysap1(-)* | 60,000 | 15,000 |
| | *Py*WT | 65,000 | 14,000 |
| 4 | *Pysap1(-)* | 63,000 | 17,500 |
| | *Py*WT | ND | 20,000 |

[1]Mean number of oocyst sporozoites per mosquito midgut dissected at day 10 post blood meal infection from ≥ 20 female mosquitoes.

[2]Mean number of salivary gland sporozoites per mosquito dissected at day 14/15 post blood meal infection from ≥ 50 female mosquitoes.

ND: not done

**Supplemental Table 2. Sequences of primers used in this study, restriction endonucleases sites are underlined.**

| Primer | Sequence |
|---|---|
| PySAP1rep1F | GGGGTACCGTGCAATGTGAAAATGATAATGCTCGATAAG |
| PySAP1rep2R | GCCCAAGCTTTTTTCTTTCTTAAATACAAAAAAATAATTTAT |
| PySAP1rep3F | GGACTAGTCCAGCTATAAACTCCGAAACATCGAATTATGT |
| PySAP1rep4R | TCCCCGCGGGCATCGCGTTGATGCTTTTGGGAATTATTGA |
| PySAP1TestF | CTCTTTTTGGGAGTCAAAAACGGTATGC |
| PySAP1TestR | CACCCTTATAACCATCATTATCTACTTTTCC |
| TgF | GGCTACGTCCCGCACGGACGAATCCAGATGG |
| TgR | CGCATTATATGAGTTCATTTTACACAATCC |
| PySAP1orfF | GGTAAACCACGGCACGTTCCTATGTTT |
| PySAP1orfR | CTTGATTTATCAGCATTGTTAATATGCCC |
| PyCSP F | AAGAAGTGTACCATTTTAGTTGTAGCGTCAC |
| PyCSP R | CACTACTGGTTGATTCAATTTATTTTGAGCCTC |
| PyTRAP F | CCAAGCAATCTACCAGAAAATCCATCTGACTCA |
| PyTRAP R | CCAGTCATTATCTTCAGGTAATTTAAACTGTTC |
| PyUIS3 F | TCATCAGGATTAGTGGCAAGTGTTATTGGGGTAC |
| PyUIS3 R | TGGTTGATATTGTTCTTTAAGAAAATGCTCCAC |
| PyUIS4 F | CACCCTGAAGTCCGAGAAAAATTTGGAATTAGA |
| PyUIS4R | TATGTATGGGTCAAATGGTTTATCATTCATACT |
| PyUIS2 F | GAAGAAGTCCCAGATGAATCAAAAGAAGATGAC |
| PyUIS2 R | ATCGTAGAAAGCTAGTTTTGTTCCGACTTTTCC |

| | |
|---|---|
| PyUIS28 F | TGGGTAGTAGTTTTGTTGAGAGAGTATGT |
| PyUIS28 R | CCAAGGATTGGGTACAAAATTGGAAGTTTCC |
| PyP52 F | AGTTGTGCATGCAAAAGAGATGAATATATCGGC |
| PyP52 R | GCCAAAATGTTCTTTTCTTTCTCAAAGGTCCATTG |
| PyS4 F | TGTTTGAGAGGCAAAAATGGATCAGAAATGTCG |
| PyS4 R | GTCATATTCTTCATCACCACCTTCATATTCCCA |
| PySPECT1 F | CCATTTTAGTCCTATTTATCATTTTAAAATGTG |
| PySPECT1 R | CAGTTTCACAACATCATCAGAAGTATCTTCA |
| PySPECT2F | GAATGTGGAACACAAGCTATGCCATTTTCTGAT |
| PySPECT2 R | TCCTATTGATTGGCCTGAACCATGTACTGAGCA |
| PyHSP70 F | CCCGATGAAGCAGTAGCATTAGGTGCAGCT |
| PyHSP70 R | TGGAACACCTCTTGGGGCTGGTGGTATACC |
| PfSAP1orfF | GAAAAATGAAGAAGATAAATCCGATGGAGATG |
| PfSAP1orfR | CGTCCTCCTTTTTATTTCGATCCTCCGTTGTAC |
| PfCSP F | GAGAAAATTAGCTATTTTATCTGTTTCTTCC |
| PfCSP R | ATCAGGATTACCATCCGCTGGTTGCTTTAA |
| PfHSP70 F | CCTGACGAAGCTGTCGCTTTAGGTGCTGCT |
| PfHSP70 R | TACACCTCTTGGTGCTGGTGGAATTCCAAC |

**Supplemental Text 1: Annotation Correction and elucidation of the coding sequence of *PySAP1***

The annotated contig for *SAP1* gene **MALPY00932** (**AABL01000929**. gi:**23491243**) contains **9721** bps and encodes only a protein of **2694** amino acids. However, comparative bioinformatic analysis with *PfSAP1* DNA and protein sequence on sequenced chromosome11 (**MAL11**, **AE014843**. gi:**23496404**) revealed that the N-terminus was missing and the C-terminus was incorrectly terminated. The missing N-terminus with the 5'UTR of *PySAP1* was located on contig **MALPY01173** (**AABL01001169**. gi: **23478694**) which contains **4359** bps and encodes a protein of **621** amino acids. These two *P. yoelii* predicted proteins overlapped identically between the first **141** amino acids of the **2694** amino acids protein (**MALPY00932**), and the amino acids **464** to **604** of the **621** amino acids protein (**MALPY01173**). We expect that a sequencing error occurred at the end of **contig MALPY01173** that caused the termination of the ORF and the splitting of the contig. In order to test this, we constructed sequencing primers where the forward sense primer (5'-TGGACTAAATAATTCTTTTCAAGTTATCGA-3') binds to a pre-overlap sequence in the **MALPY01173** contig, and the reverse antisense primer (5'-TGATAGCTATTTTGTTTAACATCTTGTTGT-3') binds to a post overlap sequence (even further downstream of the predicted stop codon of the **MALPY01173 contig**). The expected size of the amplified fragment is **690 bps**. High-fidelity Pfu DNA polymerase amplification from *P. yoelii* genomic DNA and from cDNA (amplified from salivary gland sporozoites RNA) generated both ~ **690** bps DNA fragments that were sequenced. The fragments gave the same sequencing results and matched perfectly the predicted full length *PySAP1* coding sequence. Furthermore, we constructed pre-ATG sense forward primer (5'-GCTAACGCATATACCTATGCCTAAGGACGTATC-3') (that binds to 5'UTR region in the mature SAP1 mRNA) and an antisense reverse primer (5'-GGGTTTTTCTTTTTTATGGTCAGATATGTCTTC-3') that binds in the coding sequence of the gene downstream the start codon. This sequence was amplified from salivary gland sporozoite cDNA with the expected fragment size of **574bps** and sequencing verified the matching of this sequence with the previously obtained data. For the verification of the C-terminus sequence and the intron - exon organization in this region, we constructed a forward sense primer (5'-CGAGGAAGACAAGTTCAACAATCTTTTAATCAC-3') that binds to a sequence before the end of the first exon, and an antisense primer (5'-ATGTAACGAGGTGTAGAAAAATGTCTAAA-3') that binds to a 3'UTR region in the

mature *SAP1* mRNA. Interestingly, the amplification from salivary gland sporozoite cDNA and from genomic DNA gave rise to two different fragments in size, 800 bps and 1041bps, respectively. Sequencing of both fragments revealed the correct missing exon 3 sequence, and the universal splicing signals gt and ag were detected in the fragment amplified from genomic DNA at the boundaries of the second intron separating the second exon from the third exon. Furthermore, using blast searches with the *P. yoelii SAP1* protein sequence, we retrieved the *P. berghei* **contig 4855** (**11,703 bps**) from the Welcome-trust Sanger institute blast server. In this contig we identified *PbSAP1* nucleotide sequence with the same exon-intron oragnaization, the identical splicing signals and similar intron sequences to *PySAP1* nucleotide sequence. The predicted *Py*SAP1 and *Pb*SAP1 whole protein sequences shared up to 88% amino acid identity. Prior to the assembly of the new **contig 4855**, *PbSAP1* sequence was dispersed over 2 contigs with missing end and intermediate DNA sequences; genomic sequence **PB_RP1438** (**CAAI01001256**. gi: **56490746**) and genomic sequence **PB_RP0468** (**CAAI01000407.** gi:**56493516**).

***PySAP1* coding sequence:**

ATGAGAAAAGGTAAACCACGGCACGTTCCTATGTTTTTGAGAAGCGAAAACAAAAAGATA

CTTTTTTCCTTTTTTTCATATTTTATGTCCAAAAATGATATACAATTTATTTTCGATCAT

TACGACATTGGTTTAATAGTTCTTACAAAAATACATAACATACGAAAAAGATATGAAGAA

GATAAAAATAATTTACAACATGAAAATAATAAAAATGATTTAGAAGACACAGAATCGATA

GATGAAAAAGTAAGAGCTTTTTTTTTAAACTATAAAATAAATTCGAATGAAGACATATCT

GACCATAAAAAAGAAAAACCCGAAAAAAATAAAAAAGGCAAAAAGGGTTCAGTAAACAAG

GAAAAAAGTAATGAAACTAATGAAAAAAAAATTTACTGCAAAAAATAATTATTCTATTATA

ACACGTTCAAAGACCGAGGAAATTGATCCTCTCAAGAATGATGAAAATGATAGTCCTAAT

AGTAATAATGCTCATGAAGAAAATAAACCACAAAATGAAGACATCACAAATACAAATAGC

TATCATAATAATAATGACAATCACTCCAACAATACGTCTGATATCCTCAAACAAACAGGG

CATATTAACAATGCTGATAAATCAAGAGTAGAAGGTGTAAAGCAAGAATCATGTGAAACT

ATTAGTGAATCAGGAGAATCCAATACACTAGATAAAAAAAACATTAATATTTCAACTAAA

GAAATTTGTAAAAATGTAAATAATAATAATAACACATTGAGTATTGATGAAGAAATAATC

GATAAAAACAATAATTTAAACGACCAAAATTTTGAAAATGCAGAGATTAAAAATAAAAAT

AATATAAAAACGTGTATTAATGAAACCAAAATAGAAGATAAAAATGGCCAAAAAGAAGAT

AAAAATATTATTAATAAAAATAAAGCTGACAATACAATATTAAGTAAACAAGTGGAAAAT

AATAATACAGAGCAAGAATACACAAAATTTCAAGAAGACATCTCTATGTTAATAGAAAAT

AATAAAAACGAAACTTGTAAAAATAAACTAAATTCTGAAGAGAGAAATAATACAAATGGA

GAAACCCAAAAAGAAAAAGAATTAGATGATATATTATTTCCTGTACGAAAAAAAAAATGAA

ACTTATAAAAAATTTAGTAAAAGAATTAATAAAACAAAAACTAATGAATTTATAAATTAC

CATGATGATCATTGTTTTTATATAACTGATTTATGTAGAAAAGGTACTATGTTGTCATTA

AGAAAAAATGAGGTATTAAATAAAAAAAATGCTAAAAATGCTCATTCTCACCATAAATTT

ATTAATTCATTTGGACTAAATAATTCTTTTCAAGTTATCGATGACGTTCAATCATTAAAT

AATATGTATATGAACTTAAAATTGTTTGATGGGCAATTAAATGGACAAATTAATGAAAAC

AATCTTAATGATCTCACAAATTTATTAAAATCAAAAAATAAGGCACCCTTTCACATCAAT

AATAGTGCCAATAATAACATTAGTAGTAATACAAATTCGTATATTTCATCTAATGATAAT

TTTAACATACCATCATCGTATTTTATATATGATTATACCAATACCGACAAATTTGCGTAT

ACATTAAATAACGTAGAAAATTTAGAACATATACATAATTATCAAAATGTTCATAACAAT

TTTTATCCCACTAGCCCAGAGTACACTAGCAATAACAACATTAATAATAACACTTTTATA

AGAAATTCGTTTAATAATAAAAACGATGCCGTTAACTTTAATATTAATCAAAATATTGAT

AACTGTTCTAATAAAAGTAAAACAGATATAAAAGATGAGCTCAATTCTACCCCCCTTTAT

TCCAGTAATTATCAACCAACAGTTACAACCTTCAACATTTATAAAAAGACATATTTAAAA

CCCGAAAAAACTAAAAATTATTCTAAAAATGGAAATACATTTAAAGCACAAACTGATGAT

TATATTATAAAACAACAAGATGTTAAACAAAATAGCTATCAAAATTTTTATATTAATCAA

ATCAAAAATGAACATATAACAAACACTATTAATAGCCAAAATAATATAAAAAATGTTAAC

TTCCATTCAAATAATATACCTCTGATAAAAAATATGCCCAACGGTTGTCTATTCTATCAA

AATGACACATTCGGAAACACCATCCACAATAATAGCTATAATAATGATATAAATGCTGCT

ATATCTTCTGCTACTACTTCTAGTATTGCCATCCCCACTGCTACTAATGCTAGTATTTCA

TCACCCACTTCTACTACTATTAATATTGATCAATGCTATAACCAGATATTTGAATCAAAT

GGAATAATTCATAGCAATCCCATAAGTAATAACGATTCAAATAATAATATGGTAGATTTA

AACCATATAGATCATAAAGCCAAGAATATAAATATGACATGGGGGGATCATAATTCAAAA

AATACTCACAACCATCCGAATTTAAATATCCAAACAGAGACAACAAGTATTAATAATGAT

TATTATCACACAAACAACCCTGATATAAACGAAAATTCTAGTCTGCACACTGACGAAGAA

AATAATCTTGAAGATAATAACAATATGGAAAATGCTTCTTGTTTAAAAAAAGGCATAAAT

AGAAAATGTTATTCAAATAATAGCACCATCAACAATAATAATAACAGTGATAATAACCCT

GAAAATAGAAACCAAAACGAATATAATGATTCTTGTGATGGTCAAAACAATTATTGTCAG

CAAAATAATAATAATGATGACAACAATTACGATAAAAAATATTCCGCAACTTATATCAGA

AGTAATAATTATTCAAACGGAAATATATATGACGATATTAATTATGAAACCAAGCAAAGT

GAAGGTATTATTTCAAAAAATCAAATGAAAAATAATTGTTTTCGCGATAACAATAAATTT

AACCCAACTGATTTTAAAGAAGTATCCCCTAAATCCACAAGCATTAATAATATCAGTAAC

AGCATGACATCTAATGATAGCTCTATGAATCAATATAATAATGCCAATTCCCGTTGCAAC

AACAGTGTTGGAAATAATAATACAGATTCTAATCAGTTCAAACAAAATATCAATAGATAT

AATAATAGCAATTCCATTGAACTTAAAACTAACTCACAATTAAATGATTATTCTAGTAAT

TCCAACACAATCCTTTTAATGGATGAATGTGATTCAAATGAGCTTTTAAATCCTCATAGT

AGTGATAATAATTATTTAAATGATAAAGATTTATTACATAGTAATAATATGACTCGAAAT

TGTTCTTATAATAATGATAACAGATCCAATACATTTAATGAATTGGTTAACAACATGTTA

TTTCAAAATGATGGTTCTCCTCAATCTTCAAAAAATGTAATAAAAAATTATAGCACTATA

TATGAGTCTCAAACAAATAAATTAGAAACAGTTTTTCCCAATGTACAAAATAATAATAAA

TATATGCGCCAAAATAATATACACCAGACTAATGATACAACCAGAAATGGTATAGAACAA

TTTGAATGCATTCACAATAGTGATAATTACTATATCAATGATAATAACGTTAGCAATACT

AGTAATGTTAATGATAGCAAAAATATCAATAGGGGAAACAATTCTGAAGATTTCAAAACG

ATAAAAAATGAAATAAAATATCCAGCTATAAACTCCGAAACATCGAATTATGTATATAAC

CCTCAAAATAATATAATGTATATACAAAACACCTCTGAGCAAATTACCAAAGGAAATAAT

AATAGTTATATACAATATAATACAACATATTCAATTAATAATGAAAATATAAGCACAAAT

TATTTAACCATGAATTCATTAATTAACGATCAGAATAAATTGGAAAATTGCTCAAAAAAT

AAAAATAATGTAAATGATAGTAAAATAAATTCCATATATGATGAAAAAAATCAAAAAAAT

TGGGATAATACAAGTATACCAATTAAAAATAAGCATGAAAATAACATTTATGAAATAAAA

GAAAACGATNTAAATTCTCAAGAAATCGAATTAAATGATAAAACCACCCAACATTTTGAA

AATACTGACAAAGAGGGCAATGAAAATAATTCTGATAATAATGGCAATCCTAATTTTGTA

AACAAAAATATAGAAAATAATGAGAATATAGCTAATTCCATTGGAAAAGAACAAAATTAT

ATTTTTCAGGAAAATCTCCAACATTTAGACACAAAAAATGTTAAAAACAATGAAACTTAT

TGCTTACCAAGTAATGAAATGTGTCAGGTTTCTCAAAAGCAAAACAATAACATACATGTA

TATGACGAAAAAAATATAGAAGAAAATCAAACTGTTATGTTTCAAAATAATTTACAATAT

AATCAAGCACAACCATACTATCAACCAAAAACAAAAGGTATTTATGATGATAAAACAAAT

TTTAACCCAGTGCAAAATACCAATGATTTACAAAGAAATACCAATAACATAATAACTAAC

GATCCTTTGATTAAAAACATTTATGAAAATAAAAATGCTTCTTTTATAAACTCAAAACAA

TCTGTGATATTACCCAAAAATGATGATCTTGATAATTCTAATTTTAAAGGTAATAATGAA

AATAATAGCAAAACGAAAAATATTAATGAATTTTCATTAAATAAAAATTATAAAAACAGT

ATCAATAATTCCCAAAAGCATCAACGCGATGCAGATATTAATTGTTATGATATAAATACA

AAATACAAGGAAAATTTGAAAAAACAAAAAAATAGCATCGTTTCAACTGTTGATAAAAGT

GATTATTTTGATGTTGAAAATAATGAAATTCATGGAAAAGTAGATAATGATGGTTATAAG

GGTGCAGAAATCGATAAGCAAAATAATCAAGAAATGAATAATAATTTTTGTAATAGAACA

AAATATATCAAAGTAGAAACTATAACAGACTCAATTGACGATAAAAATAACAATTCTATA

TCTTCTATTCGAAATAATACTAATAAAACCATATATGATATATATATGAATGACACTATG

CAATTAAACGAAAACATAAATAATAATAAAAACCTAAACATTCAGAACAATAGTAATGAT

ATAATAACTAAGGACAATAACACAAATAATTTGGGAAATATTGATAATAATTTTACAAAT

TCAGAATATTCACAATTTCATACGAATAATAGCGTTAAAACGCAACATAATCCAGAGCTA

TCGCAGCGCGAAATGAATTATTTATATACGAACAAAATGGCGCTTATAAAAGACAATATA

AACATAGATGCAAATGATAATATTATAAATTTACAAAACTTTAATTATATAAATGACCAA

ACAAATGATAATAAAAACAGAAAAATGAATTCCTTAACCATGAAAATGCATAGCAATGCA

TCTAGCTATATTTTTGACAATAAAACAAATAATAATAGTGAAGAACAAAAAAATAACGAT

TTTAATTTGGGCTTAAATCCTGTAAAAGATAAATATGATAATTTATACGCAAATTATTAT

GTTAATGATAATGATCACATCAATAGCAATGACCAACAAATAAAAAATTCCAACCTTGAA

ACTCAAGACACATTAAATCGAAGGAAAAAAAACACCTTAAAAACAATTTCTATGGAACAA

CTTCCATTTCACAATTCCGTGAATAATTTTGAAAATAACTACGAAAGTAATTCAAGTTGT

TATAAATATACAAAAGGCATAAACACAACCCCAATGCTAAATGAAAACATAAATAACGCA

TGCATAAACCAAGAAATTTATGATAACAATAGACAAAATATTGAAACTCCGAGATCTAAA

AATTATCCTGTATTTAACATGGGAATTCAGAATAATAAAGCAAATTCAGAAGCATTAGAT

GAAAATGGTCAAATACAATCAAAAAATGTTTTAACTAATAATTTATTTAAAATAAATAAA

AAATACAATTCAAATAATGCAGAAACAAATAACAATCTCATATATGACAATAATGAAAAT

GCTCAATATAATTATTTAAATGATTTACATACAAATGGAAGGAATTCTATTTATGAGTCG

CATATGGATTTGCCACCAAATCACCCTCATCTTTTATATTTAAATAATCTAAATGCAAAA

AACCTAGAGAATATATCTGAACGAAAAATTAGTATACCTAATGAATACATCAATTTCAAT

TCCCTTAATCATATTTACAATGTACCCCAATTTGATAATCTACCAAACAATGTAAACAGC

AATACAAGATATAATAATAATAATAATAGTAATAATAGTAATAATAGTAATATTTGTGAT

AACTCCATAAACAATACTAATAATAGCATTGACCCAAACAACAACAATACACCTACAGTG

TATAACACTGAAGGGCCTATTTCTAATGTTGATAATAATTATAATATAACAAATATGCCA

TTAGATAAAAACTCTATTATAACAAACCCTCATTATTCCAGCAACACAAATGATAATAAA

AATTCTGCAATATACAATAATATTAGATATTCAAATAGAACATTTTGCAATTATAATAAT

ATTAAAGGTTCCAATGACAACAATATTTATATTCCCAATAACAATATTAAATCATATAAT

GATAATATTATGCATAATTTGAGCAACAATGTAAAGGGTTATAATAACAGTAGTGTATAC

ACTTATCGAAATAACAATATTGCTACTAATGGAAAAAATAATAAGGAACCTAACAAAGGT

AATAATAATTTATATAATTTTAACAATTATAAAAACTATATACATAATGATTCGTCTAAT

TTAATAAATGTAAAGTCTGAACACCCTGAAAAATTTAATTATGAAAATAATTTATTAGAT

AATGTTGATAAAACAAATAAAAATAATATAAATAGGCAAATTGATAACGAAGAATTAAAC

AGTCTTATAACAGAAGCACACATGAGATCCTTTGATAATTTTAAAGCATCAGAGGAAAAT

AGTATGCGAAATAATATTAATAATAGATTTATTAACAATAATAATAATGATCATTATCAA

ATGATGATAAACAATATCAATTTATATAATGCTTATAAATATTTATATAGAAATAATAAT

AAATATATATATATAACACAAACAATAATGATAAATCATCAAATAATATAAATATTTATAGT

GATATCAATAATTTTCCTCAAAAAAAAAATTAATACGTATACACCCAATAATTTATTAACC

TCACAAAACAATAATGTGTTTAATACACAAGATAACATGCATCAAGATCAAACAAATCCA

ATTATACTATATAACAACATAAATGGAACTAAAAACAACTTAATAAGTCATATAAAAAGT

GATATACCTTCAAGTGGTTGTGATACTAATCAAAGCAATAATGACATGAAAAACGAAAAT

GGTTTTAATTATTTATATAATGGTATTCCTTTTGATTATTTCAAGAATGATATGGCTTTT

ATCTACAATAATAATATTAGAAATACTGGGAATATCATCGAAGGTACCAACATTATCAAT

GATCATAATAAACAATTTGAAAATAATGATGAACGAATGAAAGAAAATCGAAATAACAAT

ATGTATGCCAATTTAAATAACCCCTCAAATAATACAATAATTTGTAAAGACCCGAAAAAT

TATTATATTAAAAATAATTTTGATATTTTAAATAACATAGATAACTTAAATGATATAAAA

AATGAAAATAATATAAACAACAGCAATACCATAATAAATTCGTCTGTAGATTTACAAAAT

AATAAAACAAAAAATAAAGACAATCCTAATTACAATGTTAACAACTGCAACGAAAATAAT

AATAATGCTGATTTAAAATATATCCATGAATTAAATAAATTTTATAATTCAAAAGGAACA

AGTTATAAGAAAATTAATGATCGTATTAATGATACAATGAACAACATAAATATGACTAGC

AATATGAATACTACTGAAACTTTAGGAAGTAATGTAGATGATACTTACAAAAGCATTAGT

AATATAAGTACTACTAGAAATAGAAATAGTAATTTCAACATGATGAATGATAACCAATAT

ATACAGTTGCAAGACGTTGCTTATAAAATTTCGGATAATTATGATAATTCTGTAAATAAA

GAAGACACCGAATCTTGCCCCAAAAAACCAAGTAATTTAAGAAATACTAATTTAATTGAT

TCTATGAACATGATGTATTTAAATAAAGCTAATAAAAACATATACAATCATCCATATTAT

AAAAATGGTATAGACGAAAATTATTTCAATTCGAATATGAATATTTCATTACAATCAGAA

TTTAGTGACCCAAACAATATTATTCATAATAATTTGAAAAAAATAAAAACAAATAAACCA

TATAATCAGTCATATCCGTCTAACTTACCATACAATAGAAATTCTAATTATAGCAATAAT

AATATTAACGAAATAAGCCAAATAAATGAGGGAAAAAATAGAGACACAGCAAATAATTAC

TACATACCACCATATATGAACCCAAATGATCAAGCACCAGATTTTAAAGATAATTATTTG

AATACGTGCAAAAATAATATTGATATTGACAATATAAATTATTATAAGGCGCAGCAATAT

TACGCAAATAATAAGTTTATTAATGTGAATTATGACAAAAATATATATATACAAATGAACCA

AATAAGTATTCTAAAAATTACCCTAATGAAGGCTTTCGAGAAAATATAGATGGTACTTAT

AATTATAATTTGGGTATGCAAAATGAACATCATAATCCAAATTACCCATTAAATATTTTA

AATAATAACAATCTATATAATAATTATGTTTATTTAAACATGCTAAATAATGCATATAAC

ACAAACAACAACAATCTTAAATTACACAACTTAGAAGAAAATAACAAACTAATCCTATAT

AATAATAAAAAAGACACTTACACAGATTTTAACAATTCAAACCATAATGTTAACAATCCT

AATGATTTGTTTCAACTAAAAATTATGGACCCTAATGAAAATAGAATTATGAACCCCCTT

AATAATATTCAAATCCCAATTAAAAATTACTATAATCTCCAACTCTTATTAAACTATTTA

CGAGGAAGACAAGTTCAACAATCTTTTAATCACAGCGCTTCAAATACAAATAACAATAAC

AATTTACGAAGTGACACCAAAAGAAATTATTCCATATCTTCAGTAGGGTTTAGCAATCGA

AGCAAAAGGTGGACAATTTGCCCCTCGTACATATGCTGTAATAGTGCCTTGTGCCGATTC

AAATTGACATGCAATTTTAAGTTTTTACAATTCAATCATTCCAATTATAGTTTCAACATC

CCCTATGCTATATATAATTGCTACAAAAATCTTATGAACTTTTATAATTATTTTACATCA

ACACTTTGCCCTCAGCAAACCTATTTGTCGAAGCATTCTACTCAAGAACACGAAACCATA

TTCAACGTTTATTGGCATTTGCTAAACACGGAAAAATATAAATATATACAAAACATAATA

TTGTTTTTAGATAATAATTTATACACAAGAGCAGCATGGCTATTAAAAAAAGGATATAAT

ATGGATGATTTTGAAGTCCAAAAAGCAGAAAGAAAATTAATAAAACTAATGCTTTTAGTA

TTTAAATTTACTTATGACCATAAAAATGACAATTCGAAATATGAGCATATAAAGGCTTTT

CTATATCCATTAAGGAACTCCCATATAAATTTTGAAATAATGAATCGATTCTTATTTTAT

TAA

## *Py*SAP1 amino acid sequence:

MRKGKPRHVPMFLRSENKKILFSFFSYFMSKNDIQFIFDHYDIGLIVLTKIHNIRKRYEE

DKNNLQHENNKNDLEDTESIDEKVRAFFLNYKINSNEDISDHKKEKPEKNKKGKKGSVNK

EKSNETNEKKFTAKNNYSIITRSKTEEIDPLKNDENDSPNSNNAHEENKPQNEDITNTNS

YHNNNDNHSNNTSDILKQTGHINNADKSRVEGVKQESCETISESGESNTLDKKNINISTK

EICKNVNNNNNTLSIDEEIIDKNNNLNDQNFENAEIKNKNNIKTCINETKIEDKNGQKED

KNIINKNKADNTILSKQVENNNTEQEYTKFQEDISMLIENNKNETCKNKLNSEERNNTNG

ETQKEKELDDILFPVRKKNETYKKFSKRINKTKTNEFINYHDDHCFYITDLCRKGTMLSL

RKNEVLNKKNAKNAHSHHKFINSFGLNNSFQVIDDVQSLNNMYMNLKLFDGQLNGQINEN

NLNDLTNLLKSKNKAPFHINNSANNNISSNTNSYISSNDNFNIPSSYFIYDYTNTDKFAY

TLNNVENLEHIHNYQNVHNNFYPTSPEYTSNNNINNNTFIRNSFNNKNDAVNFNINQNID

NCSNKSKTDIKDELNSTPLYSSNYQPTVTTFNIYKKTYLKPEKTKNYSKNGNTFKAQTDD

YIIKQQDVKQNSYQNFYINQIKNEHITNTINSQNNIKNVNFHSNNIPLIKNMPNGCLFYQ

NDTFGNTIHNNSYNNDINAAISSATTSSIAIPTATNASISSPTSTTINIDQCYNQIFESN

GIIHSNPISNNDSNNNMVDLNHIDHKAKNINMTWGDHNSKNTHNHPNLNIQTETTSINND

YYHTNNPDINENSSLHTDEENNLEDNNNMENASCLKKGINRKCYSNNSTINNNNNSDNNP

ENRNQNEYNDSCDGQNNYCQQNNNNDDNNYDKKYSATYIRSNNYSNGNIYDDINYETKQS

EGIISKNQMKNNCFRDNNKFNPTDFKEVSPKSTSINNISNSMTSNDSSMNQYNNANSRCN

NSVGNNNTDSNQFKQNINRYNNSNSIELKTNSQLNDYSSNSNTILLMDECDSNELLNPHS

SDNNYLNDKDLLHSNNMTRNCSYNNDNRSNTFNELVNNMLFQNDGSPQSSKNVIKNYSTI

YESQTNKLETVFPNVQNNNKYMRQNNIHQTNDTTRNGIEQFECIHNSDNYYINDNNVSNT

SNVNDSKNINRGNNSEDFKTIKNEIKYPAINSETSNYVYNPQNNIMYIQNTSEQITKGNN

NSYIQYNTTYSINNENISTNYLTMNSLINDQNKLENCSKNKNNVNDSKINSIYDEKNQKN

WDNTSIPIKNKHENNIYEIKENDXNSQEIELNDKTTQHFENTDKEGNENNSDNNGNPNFV

NKNIENNENIANSIGKEQNYIFQENLQHLDTKNVKNNETYCLPSNEMCQVSQKQNNNIHV

YDEKNIEENQTVMFQNNLQYNQAQPYYQPKTKGIYDDKTNFNPVQNTNDLQRNTNNIITN

DPLIKNIYENKNASFINSKQSVILPKNDDLDNSNFKGNNENNSKTKNINEFSLNKNYKNS

INNSQKHQRDADINCYDINTKYKENLKKQKNSIVSTVDKSDYFDVENNEIHGKVDNDGYK

GAEIDKQNNQEMNNNFCNRTKYIKVETITDSIDDKNNNSISSIRNNTNKTIYDIYMNDTM

QLNENINNNKNLNIQNNSNDIITKDNNTNNLGNIDNNFTNSEYSQFHTNNSVKTQHNPEL

SQREMNYLYTNKMALIKDNINIDANDNIINLQNFNYINDQTNDNKNRKMNSLTMKMHSNA

SSYIFDNKTNNNSEEQKNNDFNLGLNPVKDKYDNLYANYYVNDNDHINSNDQQIKNSNLE

TQDTLNRRKKNTLKTISMEQLPFHNSVNNFENNYESNSSCYKYTKGINTTPMLNENINNA

CINQEIYDNNRQNIETPRSKNYPVFNMGIQNNKANSEALDENGQIQSKNVLTNNLFKINK

KYNSNNAETNNNLIYDNNENAQYNYLNDLHTNGRNSIYESHMDLPPNHPHLLYLNNLNAK

NLENISERKISIPNEYINFNSLNHIYNVPQFDNLPNNVNSNTRYNNNNNSNNSNNSNICD

NSINNTNNSIDPNNNNTPTVYNTEGPISNVDNNYNITNMPLDKNSIITNPHYSSNTNDNK

NSAIYNNIRYSNRTFCNYNNIKGSNDNNIYIPNNNIKSYNDNIMHNLSNNVKGYNNSSVY

TYRNNNIATNGKNNKEPNKGNNNLYNFNNYKNYIHNDSSNLINVKSEHPEKFNYENNLLD

NVDKTNKNNINRQIDNEELNSLITEAHMRSFDNFKASEENSMRNNINNRFINNNNNDHYQ

MMINNINLYNAYKYLYRNNNKYIYNTNNNDKSSNNINIYSDINNFPQKKINTYTPNNLLT

SQNNNVFNTQDNMHQDQTNPIILYNNINGTKNNLISHIKSDIPSSGCDTNQSNNDMKNEN

GFNYLYNGIPFDYFKNDMAFIYNNNIRNTGNIIEGTNIINDHNKQFENNDERMKENRNNN

MYANLNNPSNNTIICKDPKNYYIKNNFDILNNIDNLNDIKNENNINNSNTIINSSVDLQN

NKTKNKDNPNYNVNNCNENNNNADLKYIHELNKFYNSKGTSYKKINDRINDTMNNINMTS

NMNTTETLGSNVDDTYKSISNISTTRNRNSNFNMMNDNQYIQLQDVAYKISDNYDNSVNK

EDTESCPKKPSNLRNTNLIDSMNMMYLNKANKNIYNHPYYKNGIDENYFNSNMNISLQSE

FSDPNNIIHNNLKKIKTNKPYNQSYPSNLPYNRNSNYSNNNINEISQINEGKNRDTANNY

YIPPYMNPNDQAPDFKDNYLNTCKNNIDIDNINYYKAQQYYANNKFINVNYDKNIYTNEP

NKYSKNYPNEGFRENIDGTYNYNLGMQNEHHNPNYPLNILNNNNLYNNYVYLNMLNNAYN

TNNNNLKLHNLEENNKLILYNNKKDTYTDFNNSNHNVNNPNDLFQLKIMDPNENRIMNPL

NNIQIPIKNYYNLQLLLNYLRGRQVQQSFNHSASNTNNNNNLRSDTKRNYSISSVGFSNR

SKRWTICPSYICCNSALCRFKLTCNFKFLQFNHSNYSFNIPYAIYNCYKNLMNFYNYFTS

TLCPQQTYLSKHSTQEHETIFNVYWHLLNTEKYKYIQNIILFLDNNLYTRAAWLLKKGYN

MDDFEVQKAERKLIKLMLLVFKFTYDHKNDNSKYEHIKAFLYPLRNSHINFEIMNRFLFY
